# Craving induction through virtual reality cue-exposure for patients with alcohol dependence in rehabilitation treatment

**DOI:** 10.1038/s41598-024-81071-0

**Published:** 2024-12-18

**Authors:** N. Tsamitros, S. Gutwinski, A. Beck, S. Lange Mussons, M. Sebold, R. Schöneck, T. Wolbers, F. Bermpohl, A. Heinz, A. Lütt

**Affiliations:** 1 Psychiatric University Hospital Charité at St. Hedwig Hospital, 10115 Berlin, Germany; 2 Department of Psychiatry and Neurosciences, Charité – Universitätsmedizin Berlin, corporate member of Freie Universität Berlin and Humboldt Universität zu Berlin, Campus Charité Mitte, 10117 Berlin, Germany; 3 Institute for Mental Health and Behavioral Medicine, Department of Psychology, HMU Health and Medical University Potsdam, 14471 Potsdam, Germany; 4 Department of Psychology, University of Potsdam, Karl-Liebknecht-Str. 24-25, 14476 Potsdam, Germany; 5 TH Aschaffenburg - University of applied sciences, 63743 Aschaffenburg, Germany; 6 Salus Clinic Lindow, 16835 Lindow, Germany; 7 German Center for Neurodegenerative Diseases (DZNE), 39120 Magdeburg, Germany; 8 German Center for Mental Health (DZPG), partner site Berlin, Berlin, Germany; 9 Berlin Institute of Health at Charité – Universitätsmedizin Berlin, Berlin, Germany

**Keywords:** Alcohol dependence, Virtual reality, Cue exposure, Craving, Cybersickness, Sense of presence, Human behaviour, Rehabilitation

## Abstract

New therapeutic approaches for alcohol dependence (AD) include virtual reality (VR)-based treatments offering scalable options for cue exposure (CE), a well-established strategy in cognitive behavioral therapy. This study aimed to evaluate the feasibility and tolerability of a new VR-CE paradigm. On an explorative basis, factors influencing the induction of craving were examined. This single-arm monocentric clinical study included *n* = 21 patients with AD in inpatient rehabilitation treatment, that completed one VR-CE session including confrontation with alcohol-associated stimuli. Measurements of subjective craving before, during and after exposure, affective states, VR side effects as cybersickness and the sense of presence in VR were conducted. Craving levels during and directly after VR-CE were significantly higher than before the intervention. Craving levels 20 min after VR-CE did not significantly differ compared to those before VR-CE. Patients described a pronounced sense of presence and only mild symptoms of cybersickness. Craving was significantly correlated with cybersickness. While positive affect decreased throughout the VR exposure, negative affect did not differ significantly in pre-post-comparisons. This study shows that craving induction through our VR-CE paradigm is feasible and well-tolerated by patients with AD in long-term rehabilitation. These results contribute to the development and future research of therapeutic VR-CE approaches.

## Main

Alcohol Dependence (AD) belongs to the most burdensome health problems with an associated mortality of three million deaths worldwide^1^. Especially in the European Union, prevalence rates of AD are high with 5.2% of men and 1.7% of women suffering from this disorder^2^. Long-term rehabilitation is a recommended treatment path for patients with AD after detoxification^3,4^. It is a structured multimodal program including psychosocial interventions, e.g., therapeutic community, cognitive behavioral therapy (CBT) and skills development to promote abstinence^3^. However, while being effective, this intervention is costly and available to less than 10% of all patients with AD^5^. Given the persistent and recurrent course of AD, approximately 60% of patients with AD relapse within the first year after discharge from long-term rehabilitation^6^. New treatment options are needed to improve the outcomes of existing treatment programs, regarding abstinence rates and reduction of relapse frequency and severity.

During the last decades, digital therapeutics have gained relevance in the field of mental health care, offering easily accessible and scalable solutions^7^. Besides smartphone app technologies, Virtual Reality (VR)-based approaches have been increasingly used in clinical practice and research, both for assessment and treatment of mental disorders^8^. One promising approach is the application of VR in exposure therapies, as already being well-established in the field of anxiety disorders^9^. In the context of addictive disorders, exposure therapy is a form of CBT based on the repeated presentation of specific (addiction related) cues to induce substance craving in a controlled setting, relying on the mechanisms of habituation and extinction learning^10,11^. Since craving intensity is an important predictive factor for relapse, this approach could be an effective tool for relapse prevention^12–15^. However, exposure therapy for substance use disorders such as AD is not yet part of clinical routines. While promising, more randomized controlled trials are needed to prove the effectiveness of this approach^16,17^. Concerning the practicability of exposure therapy in clinical routines, there are still challenges to overcome, which could be efficiently addressed by the implementation of Virtual Reality: while exposure in real life requires time to visit bars or the capacity for constructing laboratory-based bars, virtual reality-cue exposure (VR-CE) offers an efficient and scalable alternative^18^. In addition to exposure therapy VR-CE environments could be also used for other therapeutic approaches, e.g., anti-craving skills and refusal skills training^19^. Technological advances in recent decades have reduced the occurrence of cybersickness, a major side effect of VR experiences, thereby increasing the tolerability of this intervention^20^. Several studies have shown that VR-based Cue Exposure (VR-CE) can effectively induce subjective craving in patients with AD^21^. Ghita et al. conducted interviews with AD patients and defined which cues were most potent in eliciting craving^22^. According to Ghita et al. being at a party, in a bar, pub, restaurant, club or being at home represent situations that lead to pronounced craving^22^. The drinks most associated with craving were beer, red wine and whiskey^22^. Participants were also asked about their current mood and they described higher craving in situations of negative emotional states such as feeling anxious, sad or stressed but also while feeling euphoric and happy^22^. Interestingly, as shown by Simon et al. “the sense of presence”—describing the feeling of being immersed in a virtual environment - seems to be another significant factor, associated with the induction of craving^23^.

The current study is part of a project aiming to develop a new VR-based exposure therapy for patients with alcohol dependence. New virtual scenarios with different alcohol-specific and contextual cues have been designed for this VR-software. As part of the development and implementation of this new VR-CE, we aim to test the feasibility of this intervention as a first step, focusing on the ability of the software to induce subjective craving as the main outcome and a sense of presence as a key element of user experience in VR interventions. Concerning the tolerability of the VR-CE, cyber sickness as a specific side effect of VR-based therapies will be examined and analyzed in association to craving and the sense of presence. Aiming to assess the general tolerability of this confrontative intervention, the affective states of the participants before and after exposure will be evaluated. On an explorative basis this study will further examine potential influencing factors determining the induction of craving, such as the duration of alcohol abstinence and time spent in rehabilitation treatment. While previous studies on VR-CE for AD included samples from non-treatment seeking populations^18,23^ or outpatient clinical settings^22,24^ the current study will focus on patients in long-term rehabilitation treatment. By including patients with AD in long-term rehabilitation, we consider a highly treatment-committed population in a vulnerable phase for relapse, constituting the target group of the planned VR-therapy.

## Results

All 21 participants completed the study. No missing data were reported.

Sociodemographic characteristics are shown in Table 1.

**Table 1:** Characteristics of sample (*n* = 21).

Male	14 (66.7%)
Female	7 (33.3%)
Age	45.2 (5.6)^a^ 24–65^b^
Education years	12.2 (1.39)^a^
Residential situation	
Flat	16 (76.2%)
Room in a shared flat	1 (4.8%)
House	4 (19.0%)
Marital status	
Single	11 (52.4%)
Married	5 (23.8%)
Separated	2 (9.5%)
Divorced	2 (9.5%)
Widowed	1 (4.8%)
Duration of abstinence (in weeks)	6.9 (5.0) ^a^
In rehabilitation since (in weeks)	1.7 (0.9)^a^

Frequency (Percent) is shown unless otherwise stated.

^a^Mean (standard deviation).

^b^Range.

Table 2 shows the characteristics of the VR-environments as selected according to patients’ preferences and the intensity level of the cue exposure paradigm. The mean duration of the exposure was 9.67 min (SD 3 min).

**Table 2:** Selected environment, selected alcoholic drink and achieved VR-CE intensity level (*N* = 21).

Selected environment	Living Room	16 (76.2%)
	Wine bar	3 (14.3%)
	Pub	2 (9.5%)
Selected alcohol drink	Beer	9 (42.9%)
	Vodka	6 (28.6%)
	White wine	4 (19.0%)
	Schnaps	1 (4.8%)
	Red wine	1 (4.8%)
VR cue exposure intensity	Level 1 + 2	11 (52.4%)
	Level 1 only	10 (47.6%)

Frequency (Percent) is shown.

**Fig. 1:**
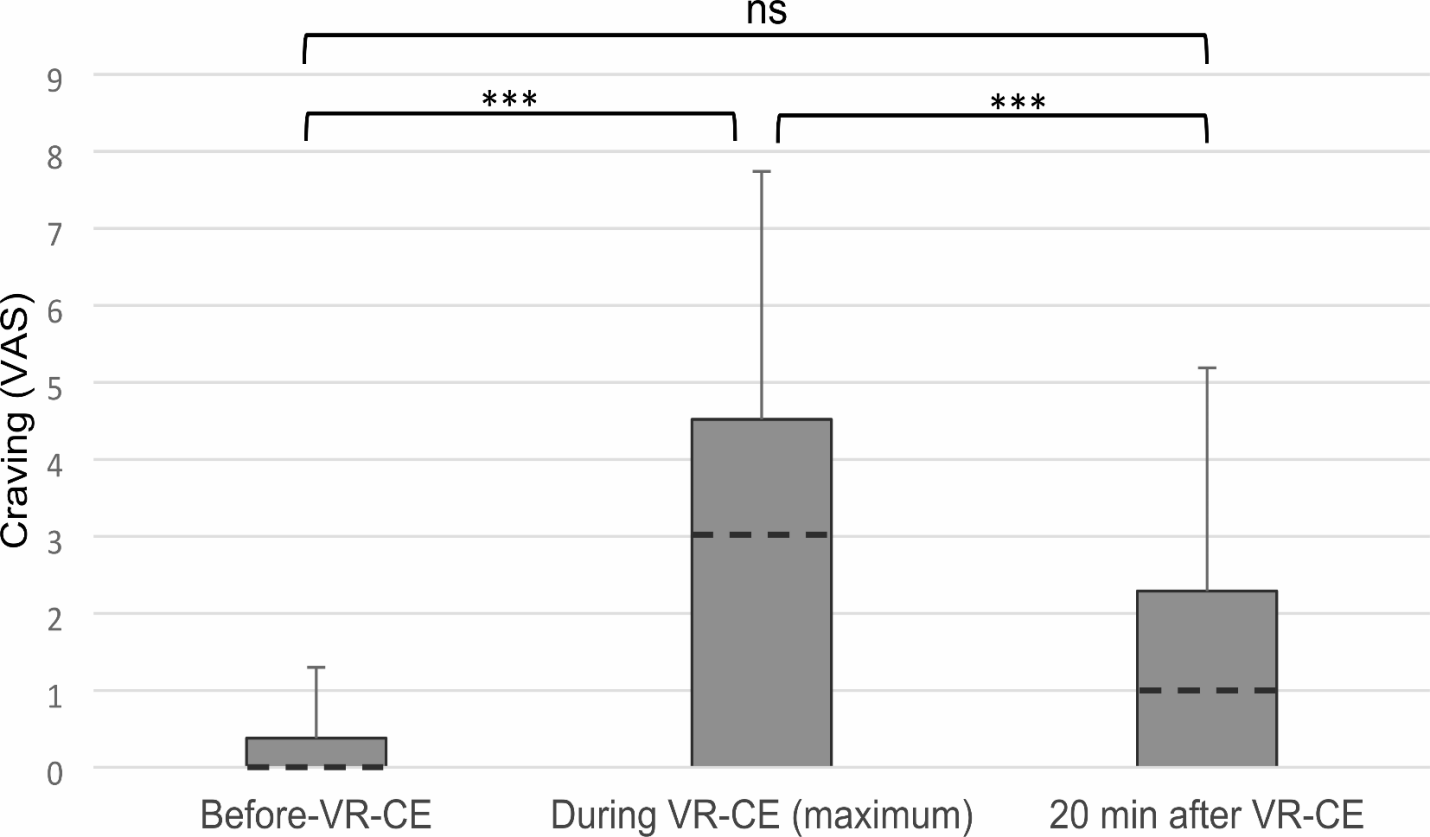
Craving measured by visual analogue scale (VAS), before VR-CE, during VR-CE (maximum), 20 min after VR-CE. Bar graphs of mean with median (dotted lines); ns = non-significant sign test pairwise comparison; * = *p* ≤ .05; *** = *p* < 0.01.

19 of 21 participants (90.5%) reported an increase of craving as measured by a visual analogue scale (VAS) during VR compared to pre-exposure. Two participants (9,5%) reported consistently very low craving and discontinued after 5 min according to study procedures. No participants required any additional psychological support during the study. Towards the end of the study, all participants reported that their current level of cravings was “tolerable” and that there was no acute risk of relapse. The comparison of craving measurements using VAS before, during (at maximum), and after VR-CE was analyzed using Friedman’s test, revealing statistically significant changes (χ² (2, *N* = 21) = 33.8, *p* < .001, W = 0.81) across the different measurement time points. Post-hoc comparisons using the Dunn-Bonferroni test (Fig. 1) showed a significant increase in craving levels between the VAS measurements before VR-CE (Mdn = 0, IQR = 2–7) and while at maximum during VR-CE (Mdn = 3, IQR = 2–7), *p* < .0.001, *r* = 0.79. Furthermore, there was a significant decrease in craving levels between maximum craving during the VR-CE and the craving level 20 min after exposure (Mdn = 1, IQR = 0–4), *p* = 0.004, *r* = 0.50. No significant difference was observed between the craving levels before and 20 min after VR-CE, *p* = .0.192, *r* = .0.29.

**Fig. 2:**
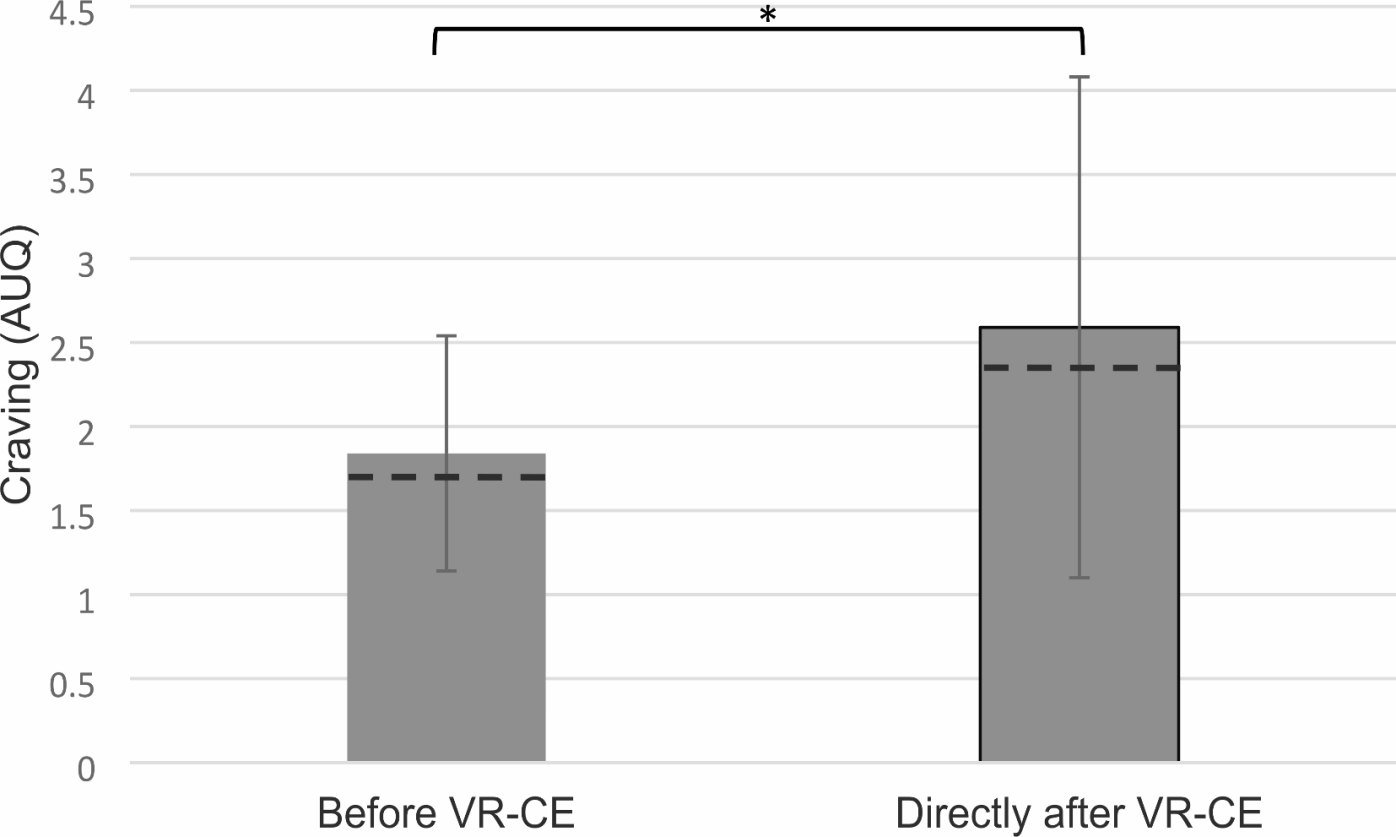
Craving measured by alcohol urge questionnaire (AUQ), before VR-CE, directly after VR-CE. Bar graphs of mean with median (dotted lines); ns = non-significant sign test pairwise comparison; * = *p* ≤ . 0.05; *** = *p* < 0.01.

As an additional measure for subjective craving besides VAS scores, the alcohol urge questionnaire (AUQ) of the whole sample showed no significant change of craving right after exposure (Mdn = 2.3, IQR = 1.1–4.0) compared to pre-exposure (Mdn = 1.8, IQR = 1.2–2.6), z(*N* = 21) = 1,66, *p* = 0.097. However, after removal of one outlier, a significant increase from pre-exposure AUQ scores (Mdn = 1.7, IQR = 1.1–2.4) to the AUQ scores right after exposure (Mdn 2.3, IQR = 1.1–4.0) was shown (Fig. 2), z(*N* = 20) = 2,15, *p* = 0.032. The removal of this value was supported by the fact that the high pre-exposure AUQ values of this participant were in contradiction with low VAS scores and probably due to the patient misunderstanding a double negative question of the questionnaire.

Table 3 shows the ratings of the feeling of presence in VR as measured through the Igroup Presence Questionnaire (IPQ). The subscales scores as displayed were evaluated according to the qualitative grading scales and acceptability rating as suggested by Melo and colleagues^25^. The total score was obtained by averaging the mean scores of all subscales and was additionally calculated after removal of one outlier on the extreme low end.

**Table 3:** Descriptive statistics for IPQ and qualitative grading according to Melo and colleagues (unless otherwise stated, *N* = 21).

	Mean		SD		Qualitative grading (A to F)	Acceptability rating
General presence	4.4		1.4		Very Good (B)	Acceptable
Spatial presence	4.6		1.0		Satisfactory (C)	Acceptable
Experienced realism	3.5		1.1		Satisfactory (C)	Acceptable
Involvement	3.0		1.3		Unacceptable (F)	Not acceptable
Total score	3.7	0.8				
Total score (without outlier, *N* = 20)	3.9	0.6				

*IPQ* Igroup presence questionnaire.

Table 4 shows the ratings of VR-related discomfort as measured through the simulator sickness questionnaire (SSQ) right after exposure. The total score represents the overall severity of cybersickness experienced by the participants^26^.

**Table 4:** Descriptive statistics for SSQ (*N* = 21).

	Mean	SD
Nausea	0.4	0.5
Disorientation	0.2	0.2
Oculomotor	0.6	0.5
Total score	1.2	1.0

*SSQ* simulator sickness questionnaire.

Positive affect as measured through the positive and negative affect schedule (PANAS) decreased significantly from pre-exposure (Mdn = 3.3, IQR = 2.8–3.6) to post exposure (Mdn = 2.9, IQR = 2.6–3.6), z(*N* = 21) = −3.0, p = 0.003. Negative affect as measured through PANAS did not significantly differ between pre-exposure (Mdn = 1.4, IQR = 1.2–2.3) and post exposure (Mdn = 1.2, IQR 1.0–1.8), z(*N* = 21) = −1.22, *p* = .0.224.

### Correlations

Spearman correlation coefficients were computed between the main outcome variable of craving and the variables of cybersickness, presence, positive affect after exposure, negative affect after exposure, abstinence length and time in rehabilitation treatment (Table 5).

**Table 5:** Spearman correlation analysis.

	Presence (IPQ)	Presence (IPQ) no outlier	Nausea scale (SSQ)	Oculomotor scale (SSQ)	Desorientation Scale (SSQ)	Positive Affect post exposure (PANAS)	Negative Affect post exposure (PANAS)	Abstinence time (in weeks)	Rehab time (in weeks)
VAS before VR-CE	0.196 (*n* = 21)	0.169 (*n* = 20)	**0.510** ^*^ (*n* = 21)	**0.457** ^*^ (*n* = 21)	0.392 (*n* = 21)	− 0.374 (*n* = 21)	0.378 (*n* = 21)	−0 0.023 (*n* = 21)	0.338 (*n* = 21)
VAS max	0.361 (*n* = 21)	0.364 (*n* = 20)	**0.583** ^*^ (*n* = 21)	**0.435** ^*^ (*n* = 21)	− 0.019 (*n* = 21)	− 0.429 (*n* = 21)	**0.440** ^*^ (*n* = 21)	0.162 (*n* = 21)	**0.439** ^*^ (*n* = 21)
VAS increase	0.329 (*n* = 21)	0.331 (*n* = 20)	**0.519** ^*^ (*n* = 21)	0.390 (*n* = 21)	− 0.090 (*n* = 21)	− 0.365 (*n* = 21)	0.367 (*n* = 21)	0.155 (*n* = 21)	0.398 (*n* = 21)
AUQ post exposure	0.148 (*n* = 20)	0.208 (*n* = 20)	**0.651** ^**^ (*n* = 21)	0.346 (*n* = 21)	− 0.164 (*n* = 21)	**− 0.597** ^**^ (*n* = 21)	**0.576** ^**^ (*n* = 21)	0.140 (*n* = 21)	**0.500** ^*^ (*n* = 21)
AUQ post exposure (no outlier)	0.151 (*n* = 20)	0.216 (*n* = 19)	**0.648** ^**^ (*n* = 20)	0.334 (*n* = 20)	− 0.179 (*n* = 20)	**− 0.604** ^*^ (*n* = 20)	**0.576** ^**^ (*n* = 20)	0.145 (*n* = 20)	**0.524** ^*^ (*n* = 21)
AUQ increase	0.314 (*n* = 21)	0.411 (*n* = 20)	**0.499** ^*^ (*n* = 21)	0.070 (*n* = 21)	− 0.218 (*n* = 21)	**− 0.610** ^**^ **(n = 21)**	0.309 (*n* = 21)	0.251 (*n* = 21)	0.336 (*n* = 21)
AUQ increase (no outlier)	0.245 (*n* = 20)	0.333 (*n* = 19)	**0.520** ^*^ (*n* = 20**)**	0.131 (*n* = 20)	− 0.202 (*n* = 21)	**− 0.558** ^**^ **(n = 21)**	0.348 (*n* = 20)	0.250 (*n* = 20)	**0.503** ^*^ (*n* = 20)

Significant values are given in bold.

*VAS*  visual analogue scale,* AUQ*  alcohol urge questionnaire, * SSQ * simulator sickness questionnaire, * IPQ* Igroup presence questionnaire, mean of total score, * PANAS*  positive and negative affect schedule.

^*^p <  0.05.

^**^p < 0.01.

## Discussion

This study shows that in patients with AD subjective alcohol craving was increased during and after VR cue exposure compared to craving levels before exposure. These findings are consistent with positive results from previous studies on the induction of craving through VR exposure and extend these results to the population of patients in rehabilitation treatment examined here^18,21–24,27–29^. In previous studies, VR-induced alcohol craving has mostly been measured using a visual analogue scale (VAS)^18,23,24,29^ or, less frequently, a questionnaire^30^. In the current study, multiple VAS ratings were used in combination with the Alcohol Craving Questionnaire (AUQ) to provide a multidimensional assessment of craving.

### Feasibility

Following the recommendations of VR clinical trials by Birckhead et al. (2019) the current study can be classified as a “VR2 trial focusing on feasibility and tolerability of the VR treatment within the intended clinical setting”^31^. In previous studies of VR-CE, participants were exposed to multiple available VR environments and drinks, either in a randomised order^18,23^, or following a graded exposure according to the participants’ rated craving hierarchy^24,30^. As this process is time consuming and may have no clear benefit, the current paradigm was based on exposure to the environment and drink that best matched the participants’ drinking habits. The induction of craving in this study indicates that the selection of individualized VR-alcohol related cues according to the patient’s preference, is appropriate for VR-based cue exposure. The most frequently selected VR environment was drinking alone in the living room. Indeed, a study on specific contexts and their association to craving, so called “highly valued contexts” in individuals with heavy drinking showed that more than half of these contexts were set at home^32^. Moreover, involuntary long-term loneliness (e.g. as experience during a COVID-19 lockdown stage) is associated with increased alcohol consumption^33^. This underlines the importance of designing VR environments for solitary drinking that are experienced by individuals with AD as of being familiar und matching their expectations.

Overall, the patients in our study reported a strong sense of presence, with corresponding IPQ scores being comparable to those of other VR therapy studies^34–36^, also particularly high on the “general presence” scale. Melo et al. developed a grading system to evaluate such scores^25^. Applying this system resulted in the best acceptability ratings with overall “acceptable” presence scores except for the component “Involvement”. Involvement can be described as the level of awareness for and engagement with the events and stimuli in VR^25^. The VR scenarios employed in this feasibility study did not include the use of controllers or e.g. direct interaction with the surrounding. Future software extensions with more interactive scenarios might lead to higher “Involvement” scores in the IPQ.

The implementation of VR-CE in rehabilitation treatment programmes would allow this intervention to be integrated into a broader therapeutic framework and enhance it according to patient needs. Patients could benefit from VR-CE by learning to perceive and rate their craving, performing repeated cue exposure therapy for habituation, adding CBT elements, or training of anti-craving skills. Depending on the treatment paradigm used, the application of VR-CE may require a more prolonged confrontation with alcohol-related stimuli or may be combined with other therapeutic techniques. However, this study focused solely on the feasibility of craving induction via VR-CE from the patients’ perspective. Offering VR-CE requires investment in hardware and software licenses but allows an easy and timely efficient application of cue exposure. For a large-scale implementation of VR-CE in real-world settings, an evaluation of its technical feasibility, organisational issues and cost-effectiveness in comparison to standard clinical treatment is recommended.

### Tolerability

The current study also focussed on potential side effects and tolerability. VR cybersickness after exposure as measured with the SSQ with a total score of < 5 is associated with only negligible symptoms and can therefore be seen as an indicator of good tolerability^37,38^. The decrease of craving 20 min after VR-CE to comparable levels as of before VR-CE indicates that the induction of craving through the intervention is transient and therefore of low-risk. This new finding suggests that VR-CE exposure can be directly applied to an individualized VR-environment that is expected to induce an intense craving response.

Positive affect decreased significantly during the intervention, but negative affect did not differ in pre-post measurements. The reduction of positive affect can be interpreted as an expected effect in the context of confrontational intervention based on craving induction and cue exposure therapy. The similar negative affect scores before and after VR-CE support the good tolerability of the VR intervention. Still, we observed a significant correlation between subjective craving and negative affect, that has already been shown in several studies^22,32^. Enabling to cope with short-term decreases of positive affect and increased craving may influence motivation and self-efficacy, which are known to be a predictors of alcohol treatment outcome^39^. The long-term effects of changes in affect should be investigated in further studies that include follow-up measures.

Given the potential for intra- and interindividual variability in craving and affective responses to VR-CE, the risks of relapse and treatment discontinuation should be assessed in future clinical trials. To minimise these risks in the current study, participants were monitored for 20 min after VR-CE.

### Influencing factors on craving

While the length of abstinence from alcohol did not correlate with induced subjective craving, craving was significantly positively correlated with the duration of rehabilitation treatment to the date of study enrolment. It could be assumed that a longer experience of the rehabilitation program could be possibly associated with an improved perception of subjective craving. This finding should be further investigated in future research.

Cybersickness symptoms as measured with the SSQ were significantly positively correlated with subjective craving as measured with the VAS and AUQ. Especially the nausea subscores showed a moderate to strong correlation with craving. One possible explanation is that patients who engaged more actively with the VR-CE environment, e.g. through 360° head movements, experienced both more craving and more cybersickness. However, since somatic symptoms such as increased salivation and sweating can be induced either by craving or cybersickness, this observation may be due to difficulties in differentiating between these sensations. The use of additional measures with questions that directly relate cybersickness symptoms to the VR experience, such as the fast motion sickness scale, and the use of cybersickness measures at different time points (before, during, and after VR-CE) should be considered in future studies with larger samples^40^.

A previous study by Simon et al. has shown that perceived ecological validity as one specific dimension of presence was related to the experience of craving during VR exposure^23^. This could not be replicated by our study as presence in VR showed no relationship with experienced craving.

### Limitations

The lack of randomization and a control group is a limitation because changes in outcomes (e.g., craving levels) cannot necessarily be attributed to the VR-CE or rather other variables may have confounded the effects of the intervention. While response bias (i.e., participants reporting increased craving because it is consistent with the goal of the intervention) cannot be ruled out by the single-arm design of the study, the inclusion of two different craving measures (VAS and AUQ) reduces this risk. Including a controlled condition group would exceed the scope of the study, that was to investigate the feasibility and tolerability of VR-CE in a specific real-world clinical setting. Future studies should also consider implementing more interactive VR scenarios using controllers to e.g. grab or reject an alcoholic drink to potentially increase involvement.

Since previous research on this topic has focused on individuals under the age of 65, and most VR-CE content has been validated for this population, this study did not include participants over the age of 65 to allow for better comparability with previous research^23,24,28,29^. Therefore, the results cannot be generalised to other age cohorts. Given the demographic shift and the limited research on VR use in geriatric contexts, further studies should address VR-CE in the elderly, as scalable therapeutic approaches are urgently needed for this group as well.

## Conclusions

The current study indicates that craving induction through our VR-CE paradigm is feasible and well-tolerated by patients with AD within a long-term rehabilitation setting. Refinements of the VR-CE paradigm aiming to increase the involvement of patients in the VR environment could further improve the VR experience. These findings can contribute to further development and encourage evaluation studies of a full-length VR-CE-based treatment procedure for this specific group of patients with AD.

## Methods

### Study design and participants

This is an experimental monocentric study employing a single-arm pre-post-test design. The targeted population included patients with AD in ongoing inpatient rehabilitation treatment at the Rehabilitation Clinic Salus Lindow, Germany. Potential participants were contacted and informed about the scope of the study by the clinical therapists. Out of the 23 individuals expressing interest in joining the study, two were ineligible for participation as they had never consciously perceived the sensation of craving. Consequently, 21 individuals, comprising 14 males and seven females (Mage = 45.2, SD = 10.6), actively took part in the research. All participants gave written informed consent and received a compensation for their participation. The study was conducted in accordance with the Declaration of Helsinki and its amendments and was approved by the local Ethics Committee of Charité—Universitätsmedizin Berlin (EA1/190/22, 23.05.2023). The current study is a feasibility study in advance of a larger study (CRAVE), ClinicalTrials.gov identifier: NCT05861843, registration date: 06/05/2023.

### Eligibility criteria

Patients were included in the study if they met the following inclusion criteria:^1^ age between 18 and 65 years;^2^ diagnosed alcohol dependence (AD) according to ICD-10 (F10.2);^3^ positive history of craving for alcohol^4^ currently at inpatient rehabilitation treatment. Patients were excluded from the study if they met the following criteria:^1^ severe neuropsychiatric comorbid disorders (i.e., psychosis, bipolar affective disorder, dementia);^2^ acute suicidality;^3^ abstinence from alcohol for less than 7 days;^4^ comorbid substance dependence other than tobacco and alcohol;^5^ concurrent treatment with benzodiazepines or anticraving medications;^6^ severe somatic disorders (e.g., photosensitive epilepsy or severe congestive heart failure).

### Measurements

In addition to the collection of sociodemographic variables, all participants completed the following questionnaires and provided information on the duration of their alcohol abstinence and time in rehabilitation treatment.

A visual analogue scale (VAS) for subjective craving: A 11-point numeric scale ranging from ‘0’ (“no craving”) to ‘10’ (“most intense craving”) was used for the assessment of acute craving directly before, during and 20 min after VR-CE. The highest VAS score reported during VR-CE was defined as the maximum craving score (VAS max). The increase of craving was defined as the difference between maximum craving score and craving score directly before VR-CE (VAS increase).

Additionally, the Alcohol Urge Questionnaire (AUQ) was used to assess craving before and directly after the VR-CE session^41^. The AUQ is an eight-item self-report questionnaire with three domains of acute craving: desire for a drink (four items), expectation of positive effect from drinking (two items) and inability to avoid drinking if alcohol was available (two items). The AUQ has shown high internal consistency (Cronbach’s alpha 0.91) as well as external validity^41^.

The sense of presence was assessed using the German version of the Igroup Presence Questionnaire (IPQ)^42^. The IPQ is a widely used self-report questionnaire to assess the feeling of presence in VR through three subscales: Spatial Presence, Involvement, Experienced Realism and one additional general item assessing Overall Sense of Presence. It consists of 14 statements rated on a 7-point Likert scale (varying from − 3 for fully disagree/not at all, to + 3 fully agree/very much).

VR-related discomfort was evaluated by the Simulator Sickness Questionnaire (SSQ)^26^. The SSQ is the most used self-reporting questionnaire to assess visually induced motion sickness, mostly defined as cybersickness. It consists of 16 items and three dimensions of cybersickness: nausea, disorientation and oculomotor disturbance. Each item can be scored on a 4-point scale (0, ‘‘none; 1, ‘‘slight’’; 2, ‘‘moderate’’; 3, ‘‘severe’’). The total score is calculated by summing up the category scores^26^.

The German adapted version of the Positive and Negative Affect Schedule (PANAS) was used for subjective mood ratings^43,44^. The questionnaire consists of 20 items, equally distributed to capture positive and negative affect.

### Study procedure

We used the “Vive Pro Eye” VR head mounted-display of HTC connected to a desktop PC with Intel Core i5-12500 and a GeForce RTX 3070 Ti graphic card. As a spatial tracking system, two wireless tracking sensors (HTC VIVE SteamVR Base Station 2.0) were used.

Participation consisted of one appointment. First, participants completed questionnaires as stated above with a standard tablet device using the REDCap software^45,46^. To achieve a personalized VR exposure close to their real-life experiences, participants were asked to choose one out of three different VR cue exposure environments and their preferred drink out of five options (see below). Thereafter, participants were immersed in a neutral VR environment (waiting room) for approximately 2 min to familiarize with the VR experience. After a confirmation that participants felt comfortable in VR the exposure paradigm was started. After the VR exposure participants completed questionnaire assessment and additionally gave a 15 min interview with open questions to acquire feedback for further adjustments of the VR content. Finally, alcohol craving according to VAS was rated again 20 min after the exposure and all participants were asked to confirm that they could safely return to their treatment schedule and without an increased acute risk for relapse otherwise an additional therapeutic support could be offered.

### Virtual reality cue exposure paradigm

**Fig. 3:**
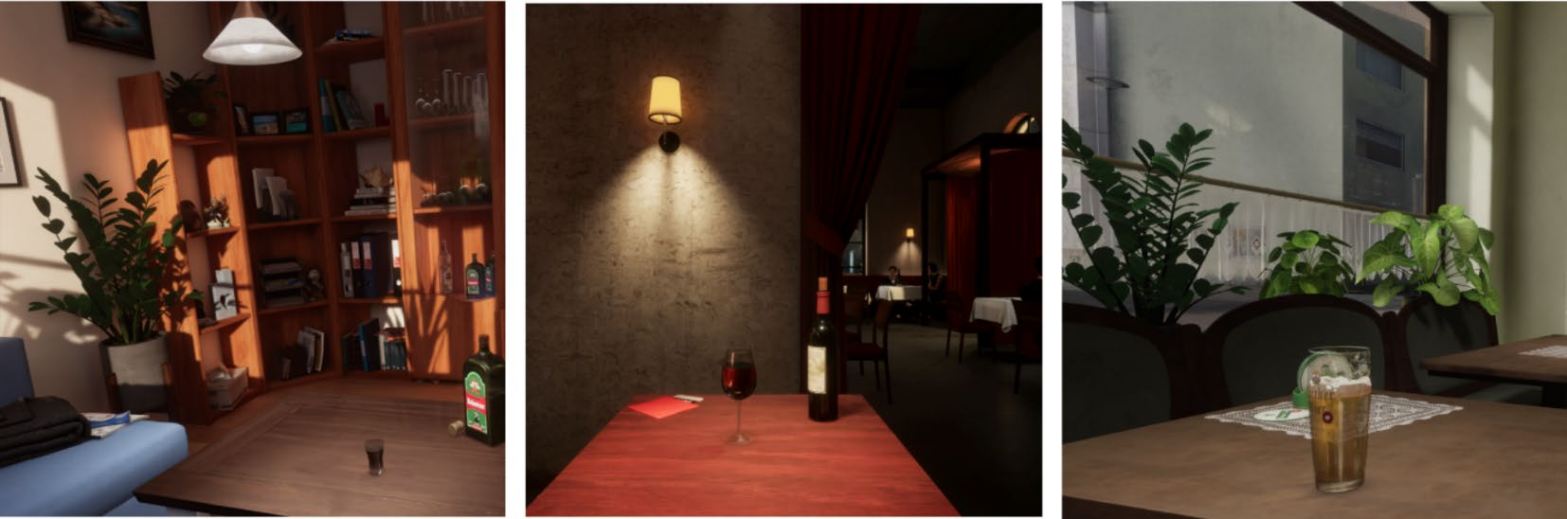
Environments of the virtual reality cue exposure paradigm (**A**) living room, (**B**) wine bar, (**C**) pub.

The VR software used in this study was developed as part of the VirtuCueR-project funded by Berlin Institute of Health (BIH) and Charité – Universitätsmedizin Berlin. The VR environments selected for this study were based on data from qualitative interviews with addiction therapists and patients as well as findings from previous research^22^. They consisted of a pub, a wine bar and a living room that enabled VR exposure to contextual alcohol-related cues, i.e., places where patients with alcohol dependence usually consume alcohol (Fig. 3). Furthermore, a selection of alcoholic beverages among beer, red wine, white wine, schnaps or vodka provided the preferred alcohol-specific cues. The participants were initially instructed to identify alcohol-related stimuli while exploring the environment and to focus on it. Participants were automatically asked to self-rate their momentary levels of alcohol craving (“how strong is your craving for alcohol at this moment?”) immediately before, 30 s after starting, and then every 90 s during the VR-CE. To avoid breaks of presence, the craving scale was integrated into the VR-environment and current craving rating could be entered using visual fixation of the according value.

To achieve an adjustable stimulus intensity, two VR-CE levels were included in the software. When craving was rated as less than 5/10 during the first 3 min (level 1), a change in the VR environment automatically occurred to intensify the VR-CE and prevent abstraction from other aspects of the VR-environment (level 2): (a) a darkening of the surrounding space and (b) focusing a light source to the alcoholic beverage in the VR environment. Otherwise, participants stayed in level 1 for the entire time of the VR-CE. The VR session had an individual duration with a predefined minimum of 5 min and maximum of 14 min. The VR-CE was terminated before 14 min when the maximum craving level as measured through VAS was reached. Thereby the intervention was discontinued if the participants showed:^1^ very low craving levels (during the first 5 min craving of 0 or 1/10)^2^ no more changes in craving levels (after the first 3 min, three subsequent ratings with the same value when craving ≤ 5/10, or four subsequent ratings when craving > 6/10) or^3^ decreasing craving levels (two ratings indicated a reduced value from the max. value).

### Statistical analysis

The statistical analysis was conducted with SPSS (Version 28). Descriptive statistics were used to characterize the sample and for all outcome variables. The normality for all sub-sets of data was tested using the Shapiro-Wilks test, with a p-value of > 0.05 indicating conformity to normal distribution. The data was examined regarding outliers. When outliers were identified the possible cause was examined and the analysis was repeated after removal. Results are presented both with and without removal of outliers. Comparison of the repeated measures of craving by VAS was performed using Friedman’s test. Post-hoc comparisons were conducted using the Dunn-Bonferroni test. For further within group comparisons the paired Wilcoxon signed rank test was used, therefore median, Interquartile Range (IQR), p and test value were reported. A correlational analysis between the craving outcomes and the other variables was conducted using Spearman’s rank correlation coefficient due to the skewness of data. For all tests, the a-priori defined significance level was *p* < .05.

## Data Availability

The datasets used and analysed during the current study can be shared by the corresponding author on reasonable request.
